# Function and activity capacity at 1 year after the admission to intensive care unit for COVID-19

**DOI:** 10.1177/02692155241262871

**Published:** 2024-06-20

**Authors:** Netha Hussain, Per-Olof Hansson, Carina M Samuelsson, Carina U Persson

**Affiliations:** 1 Department of Radiology, 56749 Sahlgrenska University Hospital, Gothenburg, Region Västra Götaland, Sweden; 2 Institute of Medicine, Sahlgrenska Academy, University of Gothenburg, Gothenburg, Sweden; 3 Department of Medicine, Geriatrics and Emergency Medicine, Sahlgrenska University Hospital/Östra, Gothenburg, Region Västra Götaland, Sweden; 4 Department of Occupational Therapy and Physiotherapy, Sahlgrenska University Hospital/Östra, Gothenburg, Region Västra Götaland, Sweden; 5 Department of Clinical Neuroscience, Rehabilitation Medicine, Institute of Neuroscience and Physiology, Sahlgrenska Academy, University of Gothenburg, Gothenburg, Sweden

**Keywords:** hand grip strength, walking speed, functional mobility, postural control, post-COVID-19 syndrome, intensive care unit

## Abstract

**Objective:**

To describe hand grip strength, walking speed, functional mobility, and postural control at one year following intensive care unit admission for COVID-19, and to find any predictors that are associated with impaired hand grip strength, walking speed, functional mobility, or postural control at the 1-year follow-up.

**Design:**

Retrospective cross-sectional and longitudinal observational study.

**Setting:**

Intensive care unit and outpatient research clinic at Sahlgrenska University Hospital.

**Participants:**

Of the 105 individuals in “The Gothenburg Recovery and Rehabilitation after COVID-19 and Intensive Care Unit” cohort, 78 participated in this study.

**Main measures:**

Descriptive statistics for hand grip strength, walking speed, functional mobility, and postural control were presented and binary logistic regressions were performed to find their significant predictors.

**Results:**

At 1-year following intensive care unit admission for COVID-19, impaired hand grip strength was found in 24.4% for the right hand and 23.1% for the left hand. Walking speed, functional mobility, and postural control were found to be impaired in 29.5%, 21.8%, and 5.1%, respectively. For impaired walking speed, longer length of stay at intensive care unit and presence of diabetes mellitus were risk factors. Diabetes mellitus was found to be the risk factor for impaired functional mobility.

**Conclusion:**

In this study, 45% of the participants showed impairment in function, activity capacity or both. These results suggest that individuals who recovered after intensive care unit admission for COVID-19 would benefit from receiving long-term follow-up to enable identification of those with need of physical health assistance and rehabilitation.

## Introduction

Recent research has shown several long-lasting effects of COVID-19, ranging from impairments of body function to limitation in activity and participation.^1^ Nearly 50% of survivors of severe COVID-19 were found to have one or more post-intensive care symptoms at 6-month post-intensive care unit admission, with 33% of the symptoms accounting for physical impairment, with a subsequent influence on the quality of life and participation.^2^

A severe COVID-19 infection is speculated to cause or amplify weakness of the skeletal muscles in affected individuals,^3,4^ and post-COVID-19 syndrome has been found to negatively impact muscle mass and function, including weakening of hand grip strength, decline in quadriceps muscle strength, and reduction in overall physical performance.^5^ Therefore, it is likely that COVID-19 has bearing on the long-term hand grip strength, walking speed, postural control, and functional mobility of individuals admitted in the intensive care unit for COVID-19.

Hand grip strength has been found to be a predictor of disease severity in individuals hospitalized for COVID-19,^6^ while those with weaker hand grip strength have been shown to be at higher risk for hospitalization due to severe COVID-19.^7^ In a recent study, hand grip strength was found to be comparable for intensive care unit-admitted individuals with and without COVID-19 at 3 months after discharge.^8^ Eight weeks after hospitalization for COVID-19, infected individuals showed lower mean walking speed and decreased functional mobility than non-infected individuals.^9^ Additionally, those with COVID-19 were found to have impaired balance at a follow-up after 4–6 months.^10^ However, there is sparse data related to hand grip strength, walking speed, functional mobility, and postural control at 1-year following intensive care unit admission for COVID-19, and describing them was the primary aim of the study. A secondary aim was to find any predictors prior to or during the intensive care unit admission that were associated with impaired hand grip strength, impaired walking speed, impaired functional mobility, and impaired postural control at 1-year follow-up.

## Methods

**Figure 1.**
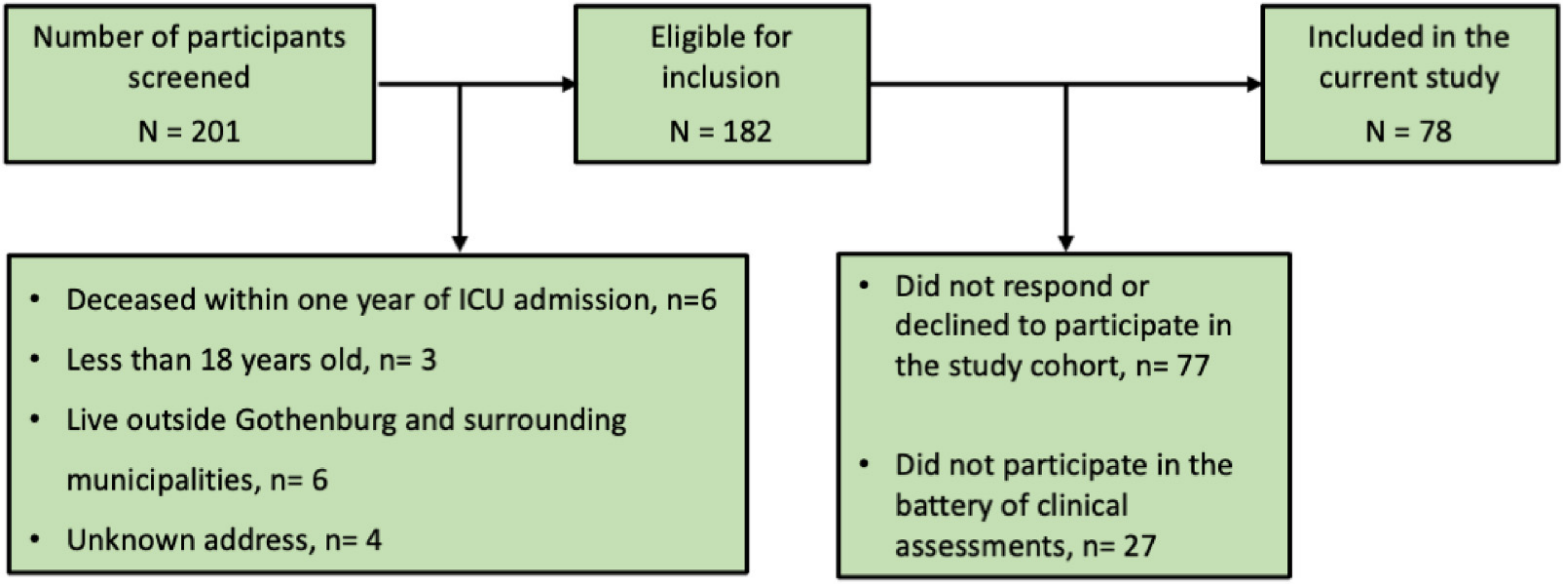
Flowchart of the inclusion process.

The participants of this study are a part of the multicenter cohort study “The Gothenburg Recovery and Rehabilitation after COVID-19 and Intensive Care Unit [GOT-RECOV-19 ICU],” with a retrospective, cross-sectional, and longitudinal observation design. This cohort consisted of 105 non-selected individuals with a confirmed COVID-19 diagnosis (i.e., with the International Classification of Diseases [ICD-10] code UO7.1) admitted to any of the five intensive care units affiliated to the Sahlgrenska University Hospital, Gothenburg, Sweden, between 01 March 2020 and 30 June 2020 (i.e., the first wave of the pandemic), and still alive 1 year after the date of admission to the intensive care unit. The inclusion and exclusion criteria of the GOT-RECOV-19 ICU cohort are shown in Figure 1. Further information regarding the cohort is presented in two previous publications.^11,12^

The participants in the GOT-RECOV-19 ICU study were invited to a follow-up visit 1 year after intensive care unit admission for COVID-19. The follow-up included assessments using a battery of questionnaires, clinical assessments, and interviews. Informed written consent was obtained from all participants before they were included in the study.

### Data collection at baseline

The demographic characteristics, comorbidities, and disease complications were collected from the medical records department (Output Data Unit) at the Sahlgrenska University Hospital. These variables included: age, sex, body mass index, comorbidities (such as hypertension, diabetes mellitus, asthma, coronary heart disease, chronic kidney disease, chronic heart failure, and chronic obstructive pulmonary disease), intensive care unit length of stay, acute respiratory distress syndrome, sepsis, and type of ventilation.

### Data collection at 1-year follow-up visit

To describe functional and activity capacity, the following assessments were performed on site: postural control, using the Berg Balance Scale;^13^ hand grip strength, using the Jamar hand dynamometer,^14^ walking speed, using the 10-meter walk test^15^ with a self-selected and comfortable speed; and functional mobility, using the Timed Up & Go.^16^ Hand grip strength and walking speed refer to function, whereas functional mobility and postural control refer to activity, according to the International Classification of Functioning and Health (ICF).^17^ Further, according to an ICF context, construct capacity is described as the highest probable level of functioning of a person in a given moment and in a standardized environment,^17^ which can represent the results from standardized assessments as in the current study.

Hand grip strength, the maximum force that can be generated by a person's forearm muscles with the elbow in 90° flexion, was assessed using the Jamar hand dynamometer,^14^ which is often considered the golden standard for measuring grip strength.^18^ For this study, the average of three measurements was taken for each hand, and both the mean strength in kilograms and the participant's hand dominance were recorded. Impaired hand grip strength was defined as having a lower hand grip strength (for either or both hands) than age- and sex-related mean reference values, obtained from a longitudinal cohort study describing normative data for hand grip strength in a community-based Australian study including 2,678 individuals.^19^

The 10-meter walk test^15^ was used to assess self-selected, comfortable walking speed (m/s) for a distance of 10 meters.^15^ It has demonstrated excellent test–retest^20^ and intra-rater reliability in healthy adults.^21^ For the current study, impaired walking speed was defined as having a walking speed lower than the 95% confidence interval (CI) compared with age- and sex-related mean reference values derived from a meta-analysis of 41 articles including 23,111 healthy adults.^22^

The Timed Up & Go is a simple, reliable, and valid screening test for evaluating functional mobility in adults.^16,23^ Timed Up & Go was found to have 87% sensitivity and specificity for identifying elderly individuals who are prone to falls.^24^ Individuals scoring less than 10 seconds were considered freely independent in their physical mobility.^16^

The Berg Balance Scale was used to determine a person's balance/postural control.^13^ It contains 14 items, each of them consisting of a 5-point ordinal scale ranging from 0 to 4, with a maximum possible total score of 56, indicating the highest level of postural control.^25^ The Berg Balance Scale is shown to have high inter- and intra-rater reliability in a meta-analysis including individuals with and without neurological conditions.^26^ A score above 45 indicates a low risk of falling. ^13^

### Statistical analysis

Descriptive statistics, using the statistical software platform SPSS (version 29.0), were presented as means and standard deviations, or medians and interquartile ranges (IQRs) for interval data. Medians and IQRs were presented for ordinal data and group size and percentages for nominal data. Mann–Whitney U test was performed to find if there are any significant differences between the participants and non-participants of the cohort in terms of age, sex, or length of stay at the intensive care unit. Binary logistic regression analyses were used to identify any factors associated with the dependent variables related to function and activity capacity. The independent variables for regression analyses were length of stay at the intensive care unit, presence of acute respiratory distress syndrome during the intensive care unit stay, presence of any chronic cardiovascular diseases, hypertension, and diabetes mellitus. For functional mobility, age and sex were included as independent variables as the cut-off score from the reference study^16^ was not adjusted for age and sex.

The level of statistical significance in the univariable and multivariable regression analyzes was defined as *p* < 0.05. To determine any predictors of impaired hand grip strength and impaired walking speed, univariable logistic regression analyses were performed first. Variables with *p*-values of <0.1, (as well as age and sex when functional mobility was the dependent variable), regardless of significance level, were included in the multivariable regression logistic regression model. Before inclusion in the multivariable regression, Spearmań's rank correlation analyses were performed to detect any multicollinearity between the independent variables, where coefficients of ≥0.7 indicated multicollinearity.^27^

The goodness-of-fit for the multivariable logistic model was determined using the Hosmer–Lemeshow test. The improvement in fit was verified using the Cox & Snell as well as the Nagelkerke pseudo R^2^ values, where higher values imply that the independent factors predict the dependent variable more accurately, but they cannot be fully understood as the proportion of explained variance. The results of the logistic regression models were presented as odds ratios (ORs), 95% CIs and *p*-values. The area under the receiver operating characteristic curve was determined for the final prediction model. An area of 70–79% under the curve was participation regarded as acceptable, 80–89% was regarded as excellent, and 90–100% was regarded as outstanding diagnostic accuracy.^28^

## Results

Out of the 182 individuals eligible for inclusion, 77 did not respond to the request to participate in the GOT-RECOV-19 ICU cohort. Out of the 105 remaining individuals, 78 (74.3%) performed the battery of clinical tests required for this study. The flowchart of the inclusion process is shown in Figure 1.

The demographic characteristics of the participants and the non-participants in the GOT-RECOV-19 ICU cohort at the time of intensive care unit admission are presented in Table 1. A large majority of the participants were male. For the 71% of the participants (n = 55) whose body mass index data was available from their medical journals via the Output Data Unit, a majority were either overweight (n = 12) or obese (n = 22), while others (n = 21) were within the healthy range. The most common comorbidity was hypertension, followed by coronary heart disease, and diabetes mellitus. Approximately half of the participants experienced acute respiratory distress syndrome, while nearly four in five underwent mechanical ventilation during the intensive care unit stay. No significant differences were found between the 78 individuals who participated in the clinical assessments and the 27 individuals who did not in terms of age (*p* = 0.46), sex (*p* = 0.46), and length of stay at the intensive care unit (*p* = 0.31).

**Table 1.** Demographic and baseline characteristics of the 105 participants in The Gothenburg Recovery and Rehabilitation after COVID-19 and Intensive Care Unit study presented by the 78 who participated in the clinical assessments (participants) and the 27 who did not (non-participants).

Demographic characteristics	Participants (N** **=** **78)	Non-participants (N** **=** **27)
	[Mean** **±** **SD, Median (IQR), n (%)]	[Mean** **±** **SD, Median (IQR), n (%)]
Age, years	56.43 ± 12.03	63.23 ± 9.77
Female/male	18 (23.1)/60 (76.9)	7 (25.9)/20 (74.1)
Body mass index (kg/m^2^) (n** **=** **55)	30.42 ± 7.74 (n = 42)	30.92 ± 8.49 (n = 13)
Comorbidities		
Hypertension	31 (39.7)	13 (48.1)
Diabetes mellitus	19 (24.4)	4 (14.8)
Asthma or COPD	8 (10.3)	2 (7.4)
Coronary heart disease	20 (25.6)	7 (25.9)
Chronic heart failure	3 (3.8)	1 (3.7)
Length of intensive unit care stay (days)	16 (8–28)	11 (7–20)
Acute respiratory distress syndrome	41 (52.6)	15 (55.6)
Minimal	2 (2.6)	1 (3.7)
Moderate	19 (24.4)	8 (29.6)
Severe	16 (20.5)	4 (14.8)
Unknown	4 (5.1)	2 (7.4)
ECMO-treated	3 (3.8)	0
Septicemia	38 (48.7)	17 (63.0)
Type of ventilation		
Mask or nasal cannula	4 (5.1)	0
High flow oxygen therapy	9 (11.5)	4 (14.8)
Mechanical ventilation (i.e., intubated)	65 (83.3)	23 (85.2)

SD: standard deviation; IQR: interquartile range; COPD: chronic obstructive pulmonary disease; ECMO: extracorporeal membrane oxygenation.

The results from the clinical assessments are shown in Table 2. Of the 78 individuals, only two (2.6%) were left-handed. Impaired hand grip strength, in comparison with age and gender-stratified normative data, was found in 19 (24.4%) participants for the right hand and in 18 (23.1%) for the left hand. Similarly, impaired walking speed compared to age and gender strata was found in 23 (29.5%) participants. Impairment in functional mobility was found in 17 (21.8%) participants, while postural control was found to be impaired in only 4 (5.1%) participants. Of the 78 participants, 43 (55.1%) had no impairments in any of the four clinical assessments, whereas 15 (19.2%), 8 (10.3%), 11 (14.1%), and 1 (1.3%) participant(s) demonstrated impairment(s) in one, two, three, or all four assessments, respectively. Sex- and age-related values for hand grip strength and walking speed are given in Tables 3 and 4, respectively.

**Table 2.** Descriptive statistics of function or activity capacity related to hand grip strength, postural control, walking speed, and functional mobility 1 year after the admission to intensive care unit due to severe COVID-19 (N = 78).

Function or activity capacity	Median (IQR) [min–max]	Mean ± (SD)	Number and proportion (%) with impaired function or activity capacity according to cut-offs for normal or age- and sex-related reference values
Hand grip strength (kg), using the Jamar hand dynamometer ^19^	Right: 40.17 (27.25–48.45) [6.4–69.8] Left: 35.85 (25.65–45.35) [3.4–61.2]	Right: 38.40 ± 13.63 Left: 35.61 ± 12.09	Right: 19 (24.35%) Left: 18 (23.07%)
Walking speed using the 10MWT (m/s) ^22^	1.24 (1.08–1.42) [0.69–1.65]	1.24 ± 0.23	23 (29.48%)
Functional mobility using TUG (s)^16^	8.28 (7.17–9.83) [4.25–13.62]	8.58 ± 2.24	17 (21.79%) (time >10 s)
Postural control, using the BBS (score) ^13^	56 (52–56) [35–56]	53.36 ± 3.93	4 (5.12%) (score <45)

IQR: interquartile range; min: minimum; max: maximum; SD: standard deviation; kg: kilograms; BBS: Berg Balance Scale; 10MWT: 10-meter walk test; m/s: meter per second; TUG: Timed Up & Go. Cut-off score of impaired hand grip strength and impaired walking speed were based on age- and sex-related mean reference values and below one standard deviation or one confidence interval, respectively.^19,22^ A BBS score >45 indicates good postural control.^13^ A TUG time of <10 s indicates independent functional mobility.^16^

**Table 3.** Hand grip strength (in kg) in the study population measured using Jamar hand dynamometer and presented as the mean value of three attempts (N = 78).

Age group (in years)	Men					Women				
	Right [mean (SD)]	^a^	Left [mean (SD)]	^b^	Total no. of participants	Right [mean (SD)]	^a^	Left [mean (SD)]	^b^	Total no. of participants
20–29	40 (8.8)	1	40 (5.6)	1	2	0	0	0	0	0
30–39	49 (2.9)	0	50 (5.8)	0	2	22 (6.3)	1	19 (5.6)	2	3
40–49	46 (12.8)	3	41 (12.2)	3	10	28 (10.5)	1	25 (8.1)	1	2
50–59	45 (13.4)	6	40 (12.0)	6	24	29 (21.4)	2	31 (17.3)	1	4
60–69	37 (11.1)	5	34 (10.1)	4	14	25 (3.3)	0	25 (4.5)	0	7
70 +	38 (7.8)	0	37 (7.9)	0	8	23 (7.4)	0	23 (9.3)	0	2

SD: standard deviation.

a Number of individuals having values below cut-off level, right side.

b Number of individuals having values below cut-off level, left side.

**Table 4.** Walking speed (in m/s) of the study population measured using the 10-meter walk test (N = 78).

Age group (in years)	Men			Women		
	Walking speed [mean (SD)]	^a^	Total no. of participants	Walking speed [mean (SD)]	^a^	Total no. of participants
20–29	1.1 (0.3)	1	2	0	0	0
30–39	1.5 (0.1)	0	2	1.2 (0.3)	1	3
40–49	1.3 (0.3)	5	10	1.1 (0.0)	2	2
50–59	1.3 (0.3)	8	24	1.2 (0.2)	1	4
60–69	1.2 (0.2)	4	14	1.1 (0.1)	1	7
70–79	1.3 (0.2)	0	5	1.3 (0.0)	0	2
80–89	1.2 (0.1)	0	3	0	0	0

SD: standard deviation.

a Number of individuals having values below cut-off level.

No multicollinearity was found between the independent variables. In Table 5, results of the univariable and multivariable regression analysis are presented for the dependent variables impaired hand grip strength, impaired walking speed, and impaired functional mobility. No significant association was found between hand grip strength and any of the independent variables. For impaired walking speed at 1-year following intensive care unit admission for COVID-19, longer stay in the intensive care unit [OR: 1.03 (CI: 1.00–1.06), *p* = 0.029] and presence of diabetes mellitus [OR: 4.63 (CI: 1.49–14.40), *p* = 0.008] at the time of admission to the intensive care unit were found to be significant predictors. For impaired functional mobility, diabetes mellitus was found to be a significant predictor [OR: 6.73 (CI: 2.00–22.68), *p* = 0.002] at the 1-year follow-up. The area under the receiver operating characteristic curve for the regression models for walking speed and functional mobility was 0.73 (CI: 0.60–0.85), *p* = 0.002 and 0.71 (CI: 0.57–0.85), *p* = 0.006, respectively, indicating an acceptable diagnostic accuracy for the two models. Impaired postural control was found in a few (5.1%) participants, so it was not considered as an outcome variable for logistic regression analysis.

**Table 5.** Univariable and multivariable analysis for prediction of impaired hand grip strength, walking speed, and functional mobility, respectively, of the participants 1 year following intensive care unit admission due to severe COVID-19 (N = 78).

Impaired hand grip strength - Univariable analysis		
Potential predictor	Odds ratio (95% CI)	*p*-value
Length of stay at the intensive care unit	1.02 (1.00–1.04)	0.108
Acute respiratory distress syndrome during intensive care unit stay	1.99 (0.69–5.71)	0.200
Chronic heart disease	0.43 (0.11–1.64)	0.215
Hypertension	0.94 (0.34–2.26)	0.943
Diabetes mellitus	1.48 (0.48–4.63)	0.497

**Table:** 

Impaired walking speed - Univariable analysis		
Potential predictor	Odds ratio (95% CI)	*p*-value
Length of stay at the intensive care unit	1.03 (1.00–1.05)	**0**.**052**
Acute respiratory distress syndrome during intensive care unit stay	1.61 (0.60–4.34)	0.344
Chronic heart disease	0.74 (0.23–2.35)	0.611
Hypertension	0.96 (0.36–2.61)	0.943
Diabetes mellitus	3.93 (1.32–11.70)	**0**.**014**

**Table:** 

Impaired walking speed: Multivariable analysis		
Potential predictor	Odds ratio (95% CI)	*p*-value
Length of stay at the intensive care unit	1.03 (1.00–1.06)	**0**.**029**
Diabetes mellitus	4.63 (1.49–14.40)	**0**.**008**

**Table:** 

Impaired functional mobility - Univariable analysis		
Potential predictor	Odds ratio (95% CI)	*p*-value
Age	1.01 (0.97–1.06)	0.645
Sex (ref = male)	0.86 (0.24–3.01)	0.810
Length of stay at the intensive care unit	1.01 (0.99–1.03)	0.304
Acute respiratory distress syndrome during intensive care unit stay	1.00 (0.36–2.83)	0.995
Chronic heart disease	0.72 (0.21–2.49)	0.599
Hypertension	2.01 (0.71–5.73)	0.191
Diabetes mellitus	6.17(1.96–19.43)	**0**.**002**

**Table:** 

Impaired functional mobility - Multivariable analysis		
Potential predictor	Odds ratio (95% CI)	*p*-value
Age	0.99 (0.94–1.04)	0.705
Sex (ref = male)	0.75 (0.19–2.94)	0.681
Diabetes mellitus	6.73 (2.00–22.68)	**0**.**002**

CI: confidence interval. For the models with outcome variables walking speed and functional mobility, respectively: Hosmer–Lemeshow (sig. level): 0.535 and 0.994, Cox and Snell R Square: 0.138 and 0.123, Nagelkerke R Square: 0.197 and 0.183. Significant *p*-values are given in bold numerals.

## Discussion

In this study, at 1-year following intensive care unit admission for COVID-19, we found that one in four participants had impaired hand grip strength, more than one in four had impaired walking speed, nearly one in four had impaired functional mobility, and 5.1% had impaired postural control. For impaired walking speed, longer length of stay at the intensive care unit and the presence of diabetes mellitus were found to be risk factors. Diabetes mellitus was also found to be a risk factor for impaired functional mobility.

Lower hand grip strength is associated with risk for general mortality and morbidity.^29,30^ Similarly, hand grip strength, measured at hospital admission, inversely predicts the risk of mortality and other poor outcomes in people with COVID-19.^31^ In our study, we found a relatively large proportion of individuals with impaired hand grip strength for both hands (19%), in relation to another study where the participants, 6 months after intensive care unit admission for various causes, reached an average of 93% of the predicted hand grip strength values.^32^

Our study also showed that 29.5% of the participants had impaired walking speed. In a similar study at 6 months following COVID-19, only 19% of the hospitalized participants had lower walking distance in a 6-min walk test.^33^ The differences in the results could be explained by our cohort being more severely sick, and the differences between the assessment methods used in the two studies.

Our results show that longer stay at the intensive care unit and the presence of diabetes mellitus were predictors of impaired walking speed at 1-year follow-up after intensive care unit admission for COVID-19. Previous studies show that those with critical illnesses can lose more than 15% of the muscle mass in lower limbs in 1 week, which can lead to intensive care unit acquired weakness and long-term detrimental effects.^34^ Similarly, the physical and psychological consequences of the intensive care unit stay and COVID-19, including fatigue^11^ may have contributed to lower walking speeds. The vascular and neurological complications of diabetes mellitus can lead to weakness, numbness, and pain in the lower extremities,^35^ which may also have contributed to impaired walking speed in the individuals of our cohort. However, we do not have information related to their neurological diagnoses or symptoms.

Impaired functional mobility was seen in nearly one-fourth of the individuals, while, surprisingly, only a minority of our participants (5.1%) had impaired postural control. A previous study of individuals with long COVID at a mean of 16 months after the illness showed that they have similar impaired balance and functional mobility as individuals with myalgic encephalomyelitis or chronic fatigue syndrome.^36^ Both groups also performed poorly compared to healthy controls,^36^ indicating that there is residual impairment in postural control and functional mobility at least up to an average of 16 months after COVID-19. Diabetes mellitus was found to be a predictor of functional mobility, which could be speculated to be due to its vascular and neurological complications. In retrospect, we found that the Berg Balance Scale, being primarily a screening instrument, did not allow us to capture any impairment in postural control in our study population.

The main strength of this study is that it not only provides a long-term follow-up of the function and activity capacity of intensive care unit-admitted individuals after COVID-19, but it is also, as far as we know, the first study to explore any baseline predictors of impaired handgrip strength, walking speed, and functional mobility. The results of this study could be generalized to intensive care unit-admitted individuals after COVID-19 during the first wave of the pandemic in Western Sweden.

This study has certain limitations. Firstly, the study consisted of 78 participants, due to which it might be underpowered to find statistical significance of some weak predictors determining the outcome. Secondly, the study design did not include a control group, which means that it is not possible to determine if the physical impairments reported in this study were caused by COVID-19 per se, by intensive care unit admission, by other medical events following the intensive care unit stay or by a combination of these. Thirdly, information for calculating body mass index, an important baseline variable, was not reported in the medical records for 23 (29%) participants, due to which it could not be included as an independent variable in the regression analyses. Fourthly, only those participants who were physically capable of attending the on-site assessments might have agreed to participate in the physical assessments, resulting in a selection bias. Fifthly, the Berg Balance Scale and the Timed Up & Go are often used to assess the elderly population, while our study group included all ages above 18 years. Furthermore, we do not have any valid data related to rehabilitation undergone by the participants following intensive care unit admission for COVID-19. Finally, as in all observational studies, the causal relationship between predictors and outcome variables cannot be established.

In conclusion, in this study, 45% of the participants showed impairment in function, activity capacity or both. These results suggest that individuals who recovered after intensive care unit admission for COVID-19 would benefit from receiving long-term follow-ups to enable identification of those with need of physical health assistance and rehabilitation.

**Clinical messages**

- Impaired hand grip strength (24.4%, right and 23.1% left), walking speed (29.5%), and functional mobility (21.8%) are present in individuals after intensive care unit admission for COVID-19 at 1-year follow-up.
- Individuals after intensive care unit admission for COVID-19 would benefit from being offered long-term follow-up for physical health assistance and rehabilitation.
